# Supplementary material for: The antimicrobial activity of silver acetate against Acinetobacter baumannii in a Galleria mellonella infection model
Source: PeerJ. 2021 Apr 22;9:e11196. doi: 10.7717/peerj.11196 (PMC8071075; doi:10.7717/peerj.11196)
Supplement: Supplemental Information 1 — Disk diffusion assays were performed according to EUCAST standards. N = 3 ± SD, NP—clinical breakpoint not available from EUCAST. Shading: Green—sensitive, yellow—intermediate, red—resistant. [file peerj-09-11196-s001.docx]

|  | Strain → | NCTC 12156 | ATCC 17978 | NCTC 13301 | NCTC 13302 | NCTC 13305 | Sensitivity | | |
| --- | --- | --- | --- | --- | --- | --- | --- | --- | --- |
| Antibiotic class | **Antibiotic ↓** | **Zone of inhibition diameter (mm ±SD)** | | | | | **S ≥**  **(mm)** | **R <**  **(mm)** | |
| Carbapenems | Imipenem | 33 (±0) | 29.5 (±0.71) | 11 (±0) | 0 (±0) | 18.5 (±0.71) | 24 | | 21 |
|  | Doripenem | 27.5 (±0.71) | 29 (±0) | 12.5 (±0.71) | 0 (±0) | 20.5 (±0.71) | 50 | | 22 |
|  | Meropenem | 30 (±0) | 29 (±1.41) | 11.5 (±0.71) | 0 (±0) | 18 (±0) | 21 | | 15 |
| Aminoglycosides | Amikacin | 18 (±0) | 17.5 (±0.71) | 3.5 (±4.95) | 8.5 (±0.71) | 0 (±0) | 19 | | 19 |
|  | Gentamicin | 12 (±0) | 19 (±0) | 0 (±0) | 0 (±0) | 0 (±0) | 17 | | 17 |
|  | Tobramycin | 17 (±0) | 19 (±0) | 0 (±0) | 12 (±0) | 10 (±0) | 17 | | 17 |
| Cephems, inc. Cephalosporins  I, II, III and IV | Ceftazidime | 23 (±1.41) | 23 (±0) | 0 (±0) | 0 (±0) | 20 (±0) | NP | | NP |
|  | Cefepime | 20.5 (±0.71) | 22.5 (±0.71) | 12 (±0) | 14 (±0) | 25 (±0) | NP | | NP |
|  | Cefotaxime | 18.5 (±0.71) | 19.5 (±0.71) | 0 (±0) | 0 (±0) | 9.5 (±0.71) | NP | | NP |
|  | Ceftriaxone | 15.5 (±0.71) | 19.5 (±0.71) | 0 (±0) | 0 (±0) | 10 (±0) | NP | | NP |
| Fluoroquinolones | Ciprofloxacin | 26.5 (±0.71) | 27.5 (±0.71) | 0 (±0) | 0 (±0) | 26.5 (2.12) | 50 | | 21 |
|  | Levofloxacin | 30 (±0) | 29.5 (±0.71) | 10.5 (±0.71) | 13.5 (±0.71) | 29 (±1.41) | 23 | | 20 |
